# Higher Dental Attainments

**Published:** 1884-11

**Authors:** J. G. Palmer


### HIGHER DENTAL ATTAINMENTS.

BY J. G. PALMER, D. D. S.

READ BEFORE THE NEW JERSEY STATE DENTAL SOCIETY.

Considering the position that New Jersey has recently assumed, and the passage of our new law in regard to the practice of dentistry, it has seemed to me that if we have any regard for the dignity of our profession, and desire to advance its interests as a whole and not as a part, there is a better opportunity than has ever before been afforded us to enhance that dignity and assist that advancement, if we will only take the necessary pains.

I take it for granted that the most, if not all, of those here to-day have a greater or less degree of respect for the profession of their choice ; that they have a certain amount of love for it, and that they revere it in proportion as it elevates and dignifies them in the eyes of the community at large. I may be speaking from my own standpoint too largely, but I should be loath to believe that there was any one here who had not a positive admiration for his chosen profession, and who would not be glad to do all in his power to advance its interests.

That such are the facts I think is proven by the presence of such numbers at our meetings, by the fact that society after society is being formed, that our membership is increasing, that our periodicals multiply, that such an increase of interest is evinced on all sides year after year.

You will ask, What has all this to do with higher dental attainments? Simply this; we must pay more attention to the ability and attainments of those whom we admit into or induce to come into our ranks. By that I mean to refer to the young men whom we employ in *any* capacity in our offices, whether as students or workmen. From their ranks are to come, subsequently, those who will take our places and upon whom we must depend to carry out whatever plans we may have laid for the advancement of our profession. We cannot ignore their claims upon us. I think that many of us have been too lax in regard to this matter. We admit to the study of dentistry those who, while bright and intelligent and quick, need more preparation in the elementary branches of their education. I do not believe that any one can know too much. I do believe that the man who passes through his collegiate course at any good classical college before he enters upon the study of his future profession, be it law, theology, medicine, or dentistry, will certainly attain a higher position than the man who has not had such good educational advantages. There may be exceptions, but this will be the rule. Then, too, such an one will have an innate regard for dignity and honor in his profession, enabling him *at once* to come to the front in any field upon which he may enter. I do not by any means intend to disparage those already engaged in the practice of dentistry, and particularly those who have never had the opportunity to qualify themselves as thoroughly as they would undoubtedly have been glad to do.

But in New Jersey, hereafter, no one may begin the practice of dentistry without a degree from some reputable dental college. I think we have in this present state of affairs a grand opportunity to advance the interests of our profession, and to add to the standing of our State Society. Let us see to it that the young men who come into our offices and under our care and guidance shall have a thorough knowledge of all that should be required of them. Let us see to it that they already have at least a common school education, before we allow them to enter our offices. Let us assist them in their studies. Then, at the proper time, let them attend lectures. When they enter upon the practice of their profession they will have higher ideas of the dignity they have to maintain, and so shall we have caused others, if not ourselves, to achieve higher attainments in our chosen walk in life.

